# Geographic and Social Factors Associated With Chronic Disease Self-Management Program Participation: Going the “Extra-Mile” for Disease Prevention

**DOI:** 10.5888/pcd16.180385

**Published:** 2019-03-07

**Authors:** Julie Bobitt, Liliana Aguayo, Laura Payne, Taylor Jansen, Andiara Schwingel

**Affiliations:** 1 University of Illinois at Urbana Champaign, Champaign, Illinois; 2 Northwestern University Feinberg School of Medicine, Chicago, Illinois; 3 Ann & Robert H. Lurie Children’s Hospital of Chicago, Chicago, Illinois; 4 University of Massachusetts Boston, Boston, Massachusetts

## Abstract

**Introduction:**

We examined geographic and social factors associated with participation in the Chronic Disease Self-Management Program (CDSMP) and the Diabetes Self-Management Program (DSMP) implemented at 144 sites in Illinois.

**Methods:**

Programs were delivered by trained facilitators, once per week, during 6 weeks to 1,638 participants aged 50 or older. Of the 1,638 participants, we included in our analysis 1,295 participants with complete geographic information and baseline data on demographic characteristics, health history, and health behaviors. We assessed the following program data: program type (CDSMP or DSMP), workshop location, class size, and number of sessions attended by participants. We geocoded each participant’s home address, classified the home address as rural or urban, and calculated the distance traveled from the home address to a workshop. We used linear and logistic regression analyses to examine the associations between participant and program factors with number of sessions attended and odds of program completion by whether participants lived in an urban or rural county.

**Results:**

Average program attendance was 4.2 sessions; 71.1% (1,106 of 1,556) completed 4 or more sessions. Most participants enrolled in CDSMP (59.6% [954 of 1,600]), but DSMP had greater completion rates. Less than 7% (85 of 1,295) of our sample lived in a rural county; these participants had better completion rates than those living in urban counties (89.4% [76 of 85] vs 75.6% [890 of 1,178]). Traveling shorter distances to attend a workshop was significantly associated with better attendance and program completion rates among urban but not rural participants. The number of sessions attended was significantly higher when class size exceeded 16 participants. Not having a high school diploma was significantly associated with lower levels of attendance and program completion.

**Conclusion:**

Participation in CDSMP and DSMP was associated with distance traveled, program type, class size, and education. Increasing participation in self-management programs is critical to ensure participants’ goals are met.

**Summary**

**What is already known on this topic?**

Two widely accepted chronic disease self-management education programs, the Chronic Disease Self-Management Program (CDSMP) and the Diabetes Self-Management Program (DSMP), give participants knowledge and skills to manage chronic diseases. However, program attendance and completion are a challenge to many program providers, and little is known about their barriers and facilitators.

**What is added by this report?**

Participation in CDSMP and DSMP was associated with distance traveled from home to workshop site, program type, class size, and education.

**What are the implications for public health practice?**

Our findings underscore the need to develop strategies to improve attendance in CDSMP and DSMP among adults aged 50 or older.

## Introduction

Management of chronic health conditions such as diabetes, hypertension, and arthritis is a public health concern among the growing older population (1). Currently, 68% of older adults have at least 2 chronic diseases (2), and people who are a racial/ethnic minority, live in a rural area, or have lower socioeconomic status are disproportionally affected (3,4). People with chronic diseases have higher risks of disability, loss of independence, and reduced quality of life, and these higher risks can lead to decreases in productivity and increases in health care costs and the burden of caregivers (5). Furthermore, chronic diseases account for 95% of health care costs in the United States (1). However, chronic diseases can be prevented and managed through healthy lifestyles and self-management education (6,7).

Two widely accepted chronic disease self-management education programs, the Chronic Disease Self-Management Program (CDSMP) and the Diabetes Self-Management Program (DSMP), give participants knowledge and skills to manage chronic diseases (7). These programs are endorsed by the National Council on Aging, and they have strong evidence to support their implementation (8). However, program attendance and completion are a challenge to many program providers, and little is known about their barriers and facilitators. Factors that influence participation are particularly important in rural areas, which often have limited access to health care services and chronic disease management programs (9). The limited evidence available about program participation in chronic disease management programs suggests that participation is greater among healthier people and urban dwellers (10,11). The objective of our study was to describe geographic and social factors associated with participation of adults aged 50 or older in chronic disease self-management programs in rural and urban areas.

## Methods

As part of a state-wide effort to disseminate and implement chronic disease self-management programs, many organizations (senior centers, Cooperative Extension offices, assisted living facilities, Area Agencies on Aging, and local hospitals) implemented the CDSMP and DSMP at 144 sites in Illinois during 2016–2017. Workshop sessions were offered once per week for 6 weeks by trained facilitators in the community’s language of preference. Participants aged 50 or older completed questionnaires at the beginning of the first workshop. The questionnaires collected information about demographic characteristics (age, sex, race, and education), health history, physical activity level, whether the respondent cared for someone with a long-term health problem or disability, and health care practices (eg, health confidence, self-efficacy in communicating with health providers). We also collected data on each participant’s home address and each workshop location.

### Measures

**Program attendance and completion.** We obtained information on attendance (number of workshops attended by participants), class size, and type of program (CDSMP or DSMP) from the workshop facilitators. The number of workshop sessions attended ranged from 1 to 6. According to program developers, attendance in 4 or more sessions is considered program completion (7). We measured program attendance as a continuous variable and completion as a dichotomous variable. We classified participants who completed 4 or more sessions as completers.

The Stanford guideline for class size requires their self-management workshops (including CDSMP and DSMP) to have a minimum of 10 and a maximum of 16 participants (12). However, adherence was low in evaluations of Stanford’s CDSMP (12). Thus, we examined adherence to class size criteria by comparing attendance and completion rates among participants in workshops that satisfied the class-size criteria and workshops that did not. We further compared attendance and completion rates for 3 workshop sizes: small (<10 participants), medium (10–16 participants), and large (>16 participants). In all regression analyses, we evaluated the effects of class size as a continuous measure.

**Geographic factors**. Geographic factors associated with program attendance and completion were whether a participant lived in a rural or an urban county and the distance traveled from a participant’s home address to the workshop site. We geocoded the address for each workshop site and participant residence into latitude and longitude by using Google Earth Pro. We used a participant’s home address to identify county of residence; we then classified these counties as rural or urban according to classifications of the US Census Bureau’s Office of Management and Budget (13,14), which defines a metropolitan (ie, urban) area as having a population of 50,000 or more and a micropolitan (ie, rural) area as having a population of 10,000 to 49,999 (13,14). We used rural as the reference category. After mapping workshop sites and participant residences by using ArcMap 10.5.1 (Esri), we calculated the distance in miles traveled by participants from home to a workshop site by using the Network Analysis tool Origin-Destination Cost Matrix. We used the dot-density function to indicate the correct number of participants per county while protecting information on participants’ exact residential locations.

To examine the effect of proximity (including on-site delivery) on attendance and completion, we dichotomized data on distance traveled by participants from home to a workshop into 2 categories: participants who traveled less than 0.1 miles (considered living in proximity) and participants who traveled 0.1 miles or more (considered not living in proximity). Participants who traveled less than 0.1 miles included participants who resided at a workshop location (residents of community housing programs, nursing homes, assisted living facilities, retirement communities, or other organizations that hosted workshops). We adopted 0.1 miles as a cut point on the basis of previous research. One study on mathematical modeling of proximity relations suggested 0.1 miles as the minimum distance for linguistic proximity analyses (15); another study used distances shorter than 200 m (approximately 0.12 miles) to set accessibility benchmarks for walking and public transportation journeys among older adults (16); and a US survey on walking for transportation that found that older adults and those with chronic diseases were more likely to favor walking short distances (17).

**Social factors.** We collected self-reported data on age, sex, race/ethnicity, level of education, and care for someone with a long-term health problem or disability. We coded race/ethnicity as a dummy variable, with non-Hispanic white as the reference category. Participants reported their education level, and we dichotomized responses into participants who did not receive a high school diploma and those who received a high school diploma or more. Participants were asked if they cared for someone with a long-term health problem or disability, and responses were dichotomized into yes or no. We assessed weekly physical activity by asking about time spent in physical activities such as walking, bicycling, and gardening. We classified answers into 2 categories to examine differences in attendance between respondents who met the national physical activity guidelines (≥150 min per week [18]) and those who did not. Finally, we collected data on class size for each workshop. 

### Statistical analyses

Overall, 1,638 adults aged 50 or older participated in CDSMP or DSMP; for 38 of these participants, we did not have information on which program they attended. We excluded 343 participants from analysis because we did not have their geographic information; our analytic sample consisted of 1,295 participants. We calculated descriptive statistics for the overall sample (those with geographic information and those without) and for our analytic sample, stratified by rural and urban residence. Not all 1,295 participants answered all questions on the questionnaire; we calculated percentages according to the number of participants who answered each question. For all available data, we conducted *t* tests to compare participants’ outcomes and identify potential cofounders. Linear regression examined factors associated with program attendance. Logistic regression examined how these factors influenced the odds of program completion. Logistic regression tested the odds of completion by using the dependent dichotomous variable to examine completion of 4 or more sessions. Independent variables were added by using the enter method, which enters variables into the model simultaneously. Both linear and logistic regression tested the influence of meeting the physical activity recommendations, living in proximity to programs (<0.1 miles), class size, type of program attended (CDSMP or DSMP), and the main effects and interaction of the miles traveled from participant’s home to a workshop, by residence in an urban or rural county. We found that attendance and completion were not influenced by caregiving, and thus we did not include this variable in our analyses. Statistical models controlled for the effect of sex, race/ethnicity, education, and program type. All analyses were computed in SPSS Statistics 24 (IBM Corporation).

## Results

**Figure 1:**
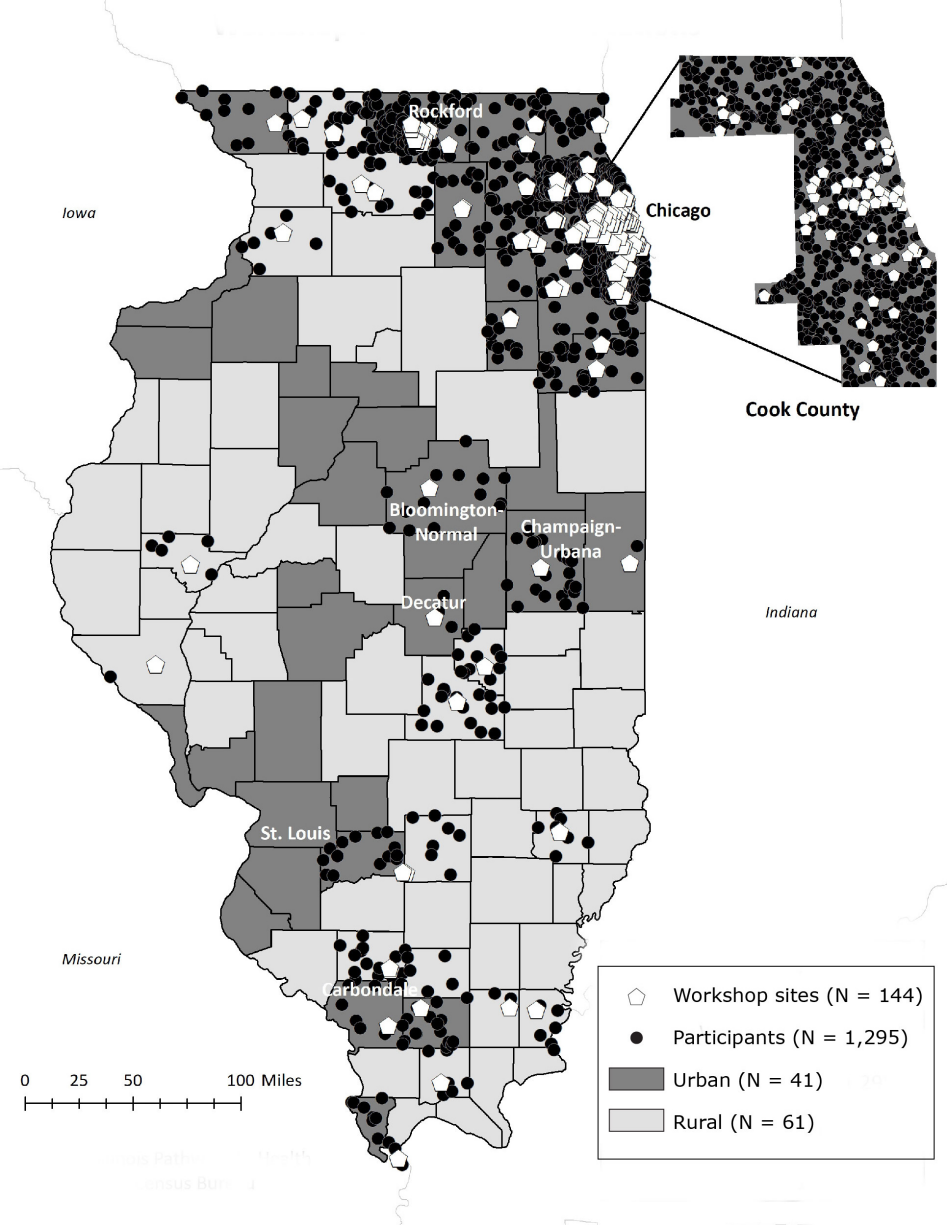
Location of workshop sites for the Chronic Disease Self-Management Program and the Diabetes Self-Management Program and distribution of participants’ home addresses by rural and urban counties in Illinois during 2016–2017. We used the dot-density function to indicate the correct number of participants per county while protecting information on participants’ exact residential locations. Data sources: Illinois Pathways to Health (19), US Census Bureau (20).

Overall, 59.6% (954 of 1,600) participants attended CDSMP, and 40.4% (646 of 1,600) attended DSMP. Of the 1,295 participants in our analytic sample, 93.4% (n = 1,210) lived in urban counties, and 6.6% (n = 85) participants lived in rural counties (Table 1 and Figure 1).

**Table 1:** Characteristics of Adults Aged ≥50 Who Participated in the CDSMP or DSMP, Overall and in the Analytic Sample, Categorized as Living in a Rural County or an Urban County,^a^ in Illinois, 2016–2017

Characteristic	Analytic Sample, by County of Residence		Overall^b^ (N = 1,638)^c^
	Urban (n = 1,210)^c^	Rural (n = 85)^c^	
**No. in analytic sample**	1,210 (93.4)	85 (6.6)	1,295 (79.1)
**Sex, no. (%)**			
Male	244 (21.4)	12 (14.5)	282 (21.1)
Female	894 (78.6)	71 (85.5)	1,056 (78.9)
**Age, mean (SD), y**	70.7 (10.6)	74.7 (7.4)	71.0 (10.5)
**Race, no. (%)**			
Non-Hispanic white	558 (52.4)	71 (88.7)	679 (54.1)
Non-Hispanic black or African American	329 (30.9)	7 (8.8)	377 (30.0)
Hispanic	91 (8.6)	1 (1.2)	107 (8.5)
Other	86 (8.1)	1 (1.2)	92 (7.3)
**Education**			
<High school diploma	122 (11.0)	5 (6.0)	148 (11.3)
High school diploma or GED	279 (25.0)	35 (42.2)	332 (25.4)
Some college or technical school	392 (35.2)	28 (33.7)	459 (35.1)
≥College graduate	321 (28.8)	15 (18.1)	370 (28.3)
**Time spent in physical activity per week, no. (%)**			
<30 min	260 (26.4)	14 (20.6)	348 (26.6)
30 min to 2.5 h	460 (46.7)	32 (47.1)	619 (47.3)
>2.5 h^d^	266 (27.0)	22 (32.4)	340 (26.0)
**Program participation**			
Enrolled in CDSMP, no. (%)	702 (58.6)	58 (68.2)	954 (59.6)
Enrolled in DSMP, no. (%)	496 (41.4)	27 (31.8)	646 (40.4)
Mean no. (SD) of sessions attended	4.4 (1.7)	4.9 (1.1)	4.2 (1.9)
Attended ≥4 sessions, no. (%)	890 (75.6)	76 (89.4)	1,106 (71.1)
Distance traveled from participant’s residence to workshop site, median (IQR), mile	2.1 (0.4–5.0)	1.3 (0.5–9.2)	2.0 (0.4–5.2)
Distance traveled from participant’s residence to workshop site, mean (SD), mile	4.1 (8.1)	4.9 (5.7)	4.9 (6.1)
Class size, mean (SD)	16.5 (7.8)	11.5 (3.6)	16.0 (7.3)
**Provides care to someone with a long-term health problem or disability, no. (%)**	319 (28.9)	16 (19.5)	370 (28.6)

Abbreviations: CDSMP, Chronic Disease Self-Management Program; DSMP, Diabetes Self-Management Program; GED, general educational development certificate; IQR, interquartile range; SD, standard deviation.

a Geographic information was available for 1,295 of 1,638 participants; only these 1,295 participants were classified as living in a rural or an urban county and comprised our analytic sample. Urban and rural classifications were determined by using participants’ home address and criteria from the US Census Bureau’s Office of Management and Budget (13,14).

b “All” participants refers to all participants in CDSMP or DSMP: the 1,295 for whom geographic information was available (the analytic sample), plus the 343 participants for whom geographic information was not available.

c Not all numbers in categories add to number in column head because not all participants answered all questions. Percentages in each category sum to 100% (unless because of rounding they do not) and are based on number of participants who answered the question.

d Satisfies current physical activity recommendations per US guidelines (≥150 min/wk [18]).

Of the 1,556 participants for whom we had attendance data, 1,106 (71.1%) completed 4 or more sessions (Table 1), and mean (standard deviation [SD]) attendance was 4.2 (1.9) sessions. Participants living in rural counties had a higher completion rate (76 of 85 [89.4%]) than participants living in urban counties (890 of 1,178 [75.6%]). Program completion and mean number of sessions attended in CDSMP and DSMP varied by workshop site. Overall, mean (SD) attendance in DSMP (4.4 [1.8] sessions) was significantly greater than attendance in CDSMP (4.1 [1.9] sessions; *t*_1,550_ = −2.9, *P* = .004). Overall, estimated median (interquartile range [IQR]) distance traveled to a workshop site was 2.0 (0.4–5.2) miles. Participants in rural counties traveled a median (IQR) distance of 1.3 (0.5–9.2) miles to a workshop, and participants in urban counties traveled a median (IQR) of 2.1 (0.4–5.0) miles. Most (88.7%; 71 of 80) participants in rural counties were non-Hispanic white; the 1,064 participants in urban counties were more racially and ethnically diverse, with 52.4% (n = 558) non-Hispanic white, 30.9% (n = 329) non-Hispanic black or African American, and 8.6% (n = 91) Hispanic. Of the 1,295 participants in our analytic sample, 28.6% (n = 370) reported caring for someone with a long-term health problem or disability.

Workshop sizes ranged from 4 to 39 participants (mean [SD] = 16.0 [7.3] participants). Overall, 41.6% (653 of 1,569) of participants attended workshops that satisfied the Stanford guideline of 10 to 16 participants. Of the 58.4% (916 of 1,569) who attended workshops that did not satisfy the Stanford guideline, 19.9% (313 of 1,569) of participants attended workshops with fewer than 10 participants, and 38.4% (603 of 1,569) attended programs with more than 16 participants. Mean [SD] attendance in small workshops (4.3 [1.8] sessions) was greater than attendance in workshops that satisfied the Stanford guideline (4.1 [1.9] sessions) (*t*_1,519_ = 2.1; *P* = .04). We found no significant differences in mean attendance between large (>16 participants) and small (<10 participants) workshops. However, mean (SD) attendance per class was higher for large workshops (4.4 [1.8] sessions) than for workshops that satisfied class size requirements (4.1 [1.9] sessions) (*t*_1,215_ = −2.7; *P* = .008).

The overall model explained 4.8% of the variance in the number of sessions attended (*R* = 0.24, adjusted *R* ^2^ = 0.048) (Table 2). Not having a high school diploma (β = −0.09, *P* = .01) and class size (β = 0.11, *P* = .01) were significantly associated with fewer sessions attended. Fewer miles traveled from home to a workshop (β = −0.12, *P* = .001) was significantly associated with a greater number of sessions completed.

**Table 2:** Linear Regression Coefficients of Variables Associated With Number of Sessions Attended Among Adults Aged ≥50 in the CDSMP and DSMP, Illinois, 2016–2017^a^

Variable	B (Standard Error)	β (95% Confidence Interval) [*P* Value]
**Sex**		
Male	−0.13 (0.14)	−0.03 (−0.40 to 0.13) [.32]
Female	Reference	
**Race**		
Non-Hispanic white	−0.27 (0.14)	−0.09 (−0.55 to 0.01) [.055]
Non-Hispanic black, Hispanic, or other	Reference	
**Education**		
<High school diploma	−0.49 (0.19)	−0.09 (−0.86 to −0.11) [.01]
≥High school diploma	Reference	
**Physical activity recommendations^b^**		
Satisfies	0.07 (0.12)	0.02 (−0.17 to 0.31) [.57]
Does not satisfy	Reference	
**Distance traveled from participant’s residence to workshop site **		
<0.1 mile	0.32 (0.16)	0.07 (0 to 0.64) [.05]
≥0.1 mile	Reference	
**Class size**	0.02 (0.01)	0.11 (0.01 to 0.04) [.01]
**Type of program**		
DSMP	0.02 (0.13)	0.01 (−0.24 to 0.29) [.87]
CDSMP	Reference	
**Classification of participant’s county of residence**		
Urban	0.16 (0.30)	0.03 (−0.43 to 0.74) [.60]
Rural	Reference	
**No. of miles traveled from participant’s home to workshop site**	−0.03 (0.01)	−0.12 (−0.05 to −0.01) [.001]
**Distance traveled × urban or rural county of residence^c^**	0.07 (0.04)	0.09 (0 to 0.14) [.049]

Abbreviations: CDSMP, Chronic Disease Self-Management Program; DSMP, Diabetes Self-Management Program.

a Attendance information for each participant in the program was provided by facilitators of the CDSMP and DSMP programs. The number of attended sessions ranged from 1 to 6. The overall model explained 4.8% of the variance in the number of sessions attended (*R* = 0.24; Adjusted *R* ^2^ = 0.048; Δ*R* ^2^ = 0.004; *P* = .049).

b Per US guidelines (≥150 min per week [18]).

c Urban and rural classifications were determined by using participants’ home address and criteria from the US Census Bureau’s Office of Management and Budget (13,14).

### Moderation between distance and attendance among participants who resided in urban counties

**Figure 2:**
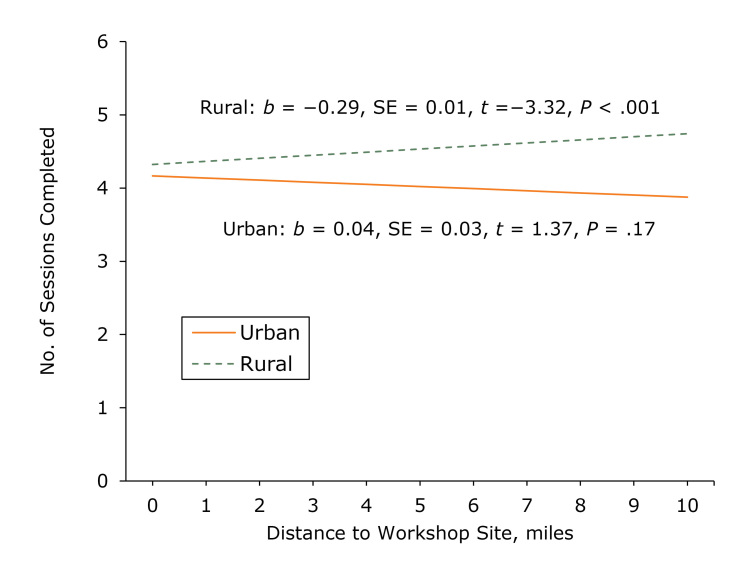
Simple slopes describing the association between the distance in miles between a participant’s home address to a workshop site and the number of program sessions completed among from adults aged 50 or older in the Chronic Disease Self-Management Program or the Diabetes Self-Management Program in Illinois during 2016–2017. We used a participant’s home address and criteria from the US Census Bureau’s Office of Management and Budget (13,14) to determine whether the participant lived in a rural county or an urban county. Abbreviation: SE, standard error.

We found a significant interaction between miles traveled from home to a workshop and whether participants lived in an urban or a rural county (β = 0.09, *P* = .049). The simple slopes showed that distance traveled from home to a workshop significantly influenced the number of sessions completed by participants living in an urban county (*b* = −0.29, *P* < .001) (Figure 2). In contrast, distance traveled had no significant effect on the number of sessions completed by participants living in a rural county (*b* = 0.04, *P* = .17).

**Table:** 

Distance Between Participant’s Home Address and Workshop Site, Miles	Participant’s County of Residence, No. of Sessions Completed	
	Urban	Rural
0	4.166	4.322
1	4.137	4.364
2	4.108	4.406
3	4.079	4.448
4	4.050	4.490
5	4.021	4.532
6	3.992	4.574
7	3.963	4.616
8	3.934	4.658
9	3.905	4.700
10	3.876	4.742

Although we did not find an interaction effect, the main effects of several geographic and social factors were significantly associated with the odds of program completion (Table 3). The overall logistic regression model explained 9% of the variance in odds of completing the program (*R* = 0.06, Nagelkerke-adjusted *R* ^2^ = .090). Participants with a high school diploma or more were nearly 2 times as likely as participants who did not have a high school diploma to complete at least 4 workshop sessions (odds ratio [OR] = 0.54, *P* = .02). Participants who traveled less than 0.1 miles to attend a workshop session were 1.69 times as likely as participants who traveled 0.1 miles or more to complete the program (OR = 1.69, *P* = .04). Odds of program completion decreased as distance between a participant’s home and workshop site increased (OR = 0.96, *P* = .008). The interaction between distance traveled by whether participants lived in an urban or a rural county was not significant (OR = 1.20, *P* = .07).

**Table 3:** Logistic Regression Coefficients of the Odds of Program Completion of the CDSMP and DSMP Among Rural and Urban Adults Aged ≥50 in Illinois, 2016–2017^a^

Variable	Odds Ratio (95% Confidence Interval) [*P* Value]
**Sex**	
Male	0.81 (0.54–1.19) [.28]
Female	1 [Reference]
**Race**	
Non-Hispanic white	0.75 (0.49–1.16) [.20]
Non-Hispanic black, Hispanic, or other	1 [Reference]
**Education**	
<High school diploma	0.54 (0.31–0.92) [.02]
≥High school diploma	1 [Reference]
**Physical activity recommendations^b^**	
Satisfies	1.26 (0.86–1.85) [.24]
Does not satisfy	1 [Reference]
**Distance traveled from participant’s residence to workshop site**	
<0.1 mile	1.69 (1.02–2.81) [.04]
≥0.1 mile	1 [Reference]
**Class size**	1.04 (1.01–1.08) [.007]
**Type of program**	
DSMP	1.27 (0.84–1.94) [.26]
CDSMP	1 [Reference]
**Classification of participant’s county of residence^c^**	
Urban	0.86 (0.31–2.40) [.78]
Rural	1 [Reference]
**No. of miles traveled from participant’s home to workshop site**	0.96 (0.92–0.99) [.008]
**Distance traveled × urban or rural county of residence^c^**	1.20 (0.99–1.47) [.07]

Abbreviations: CDSMP, Chronic Disease Self-Management Program; DSMP, Diabetes Self-Management Program.

a The number of attended sessions ranged from 1 to 6. Attendance at ≥4 of 6 sessions is considered program completion by the program developers (7).The overall logistic regression model explained 9% of the variance in odds of completing the program (*R* = 0.06, Nagelkerke-adjusted *R* ^2^ = .090).

b Per US guidelines (≥150 min per week [18]).

c Urban and rural classifications were determined by using participants’ home address and criteria from the US Census Bureau’s Office of Management and Budget (13,14).

## Discussion

Our findings indicate that travel distance was a barrier for attendance among participants who lived in urban counties but not rural counties. A study in 2014 also found that distance was a barrier to accessing health care resources more often among urban dwellers than rural dwellers (21). That study asserted that rural residents are used to navigating distances and therefore may negotiate them better, whereas urban dwellers may have difficulties finding transportation for even a short distance if they have no vehicle or have mobility issues (21). Therefore, when working with older adults, especially those in urban communities, program planners should pay attention the distance people must travel to get to program sites. Sites should be situated in neighborhoods where the target population lives. On-site delivery (delivery in senior housing programs or community housing sites where adults aged 50 or older reside) may be an option.

Rural dwellers in our study had higher rates of completion and attendance than urban dwellers. This finding is consistent with findings of a nationwide study of chronic disease management dissemination that examined data on more than 300,000 participants in rural and urban areas (9). One explanation is that health education programs may compete with other activities to which urban dwellers have access locally. In contrast, rural communities tend to offer fewer “distractions” and residents may make such health education programs their priority.

Although program coordinators reached out to rural areas through senior centers, Extension offices, AAAs, and local hospitals, rural areas were underserved. Rural areas are home to 18.6% of adults aged 65 or older in Illinois (22), yet they represented only 6.6% of our sample. Several factors, such as access to appropriate meeting facilities, affect rural service delivery (23). The availability of program facilitators and partnerships in the southern, less populated areas of Illinois was a challenge for program delivery. The workshops in rural areas were attended mostly by non-Hispanic white people. However, rural areas are more homogenous than urban areas in Illinois: only 9.5% of the population is black and 11.7% Hispanic (22). Rural areas can benefit from more culturally tailored recruitment strategies.

Attendance rates in DSMP were significantly greater than in CDSMP. The better attendance in DSMP could have been due to a more focused workshop content. Erdem and Korda reported higher completion rates for people with diabetes who participated in DSMP than in CDSMP, also attributing that outcome to the focus on diabetes in DSMP (24). The study suggested that higher completion rates could be due to factors such as type of recruiting methods, program site (ie, senior center vs health care facility), and type of program offered near participants’ home. Future research should consider administering both the CDSMP with DSMP in the same location at the same time to determine differences in attendance or completion outcomes and whether any differences can be explained by geographic factors.

Our results indicated that class size was associated with attendance. This finding suggests that the experience of participating in a chronic disease program goes beyond its content. Participation in such programs likely promotes social interactions essential to motivating and encouraging attendance. Gallant reported on the valuable role of friends in chronic disease self-management and emphasized the importance of self-management educational programs to incorporate skills and strategies that enhance social interactions (25). Meek and colleagues found that older adults with chronic disease restrict their social engagement with family, friends, and community (25). They suggested that behavioral interventions could help older adults better manage their chronic conditions and maintain active social lives (25). These findings underscore the importance of recruitment that leads to larger classes, and ultimately, increases attendance. Although some studies found that programs with fewer participants had higher attendance rates, the settings for these programs varied, and therefore the results were inconclusive (12,24). Interestingly, these studies included sites that delivered programs to classes that were larger than the class size specified in the Stanford guideline. These findings warrant further study because adapting chronic disease self-management programs to fit a particular facility could affect program fidelity, which may also affect program efficacy (27). However, Smith and colleagues suggested that some flexibility can be allowed in program implementation to fit the needs of a particular facility as long as the core elements of the program are maintained and program outcomes are not compromised (28). Both Smith and colleagues (28) and Carvahlo and colleagues (27) suggested further research to determine whether significant outcomes can be achieved when interventions are adapted to a particular environment.

Low educational attainment was associated with lower attendance in rural and urban areas. Much literature exists on the association between education and health literacy, defined as “the degree to which individuals have the capacity to obtain, process, and understand basic health information and services needed to make appropriate health decisions” (29). Older adults have a double burden, with a disproportionally high prevalence of chronic diseases and a greater risk of poor health literacy (29). In the context of chronic disease self-management, education affects a person’s ability to read health information, process oral communication, and conceptualize activities (30). Mackey and colleagues found that health literacy-sensitive interventions resulted in significant improvements in self-care practices (30). Educational barriers may be associated with a limited sense of purpose and belonging in health programs, which can create feelings of frustration and affect attendance and completion. Zoellner and colleagues underscored the importance of integrating recruitment strategies that attend to the needs of audiences with a low level of education (31). Although CDSMP and DSMP materials address low literacy levels, our findings reiterate the importance of focusing on how chronic disease self-management programs are designed and marketed. Best practices include the creation of easy-to-read marketing and communication materials with appropriate language, font style, and font size (32). Graphic illustrations and experiential activities are useful strategies in mitigating some education limitations and could result in increased attendance (32).

Our study had several limitations. First, we had a small sample of rural residents. Although we assessed distance objectively through geographic information, our assessment did not account for the time burden associated with various modes (eg, car, bus, walking) of transportation. Additional limitation was the use of only 2 measures of rurality/urbanicity (ie, rural and urban), which may not have accounted for racial/ethnic diversity among the rural population. Also, information about physical activity collected in the questionnaire did not include information on the intensity of physical activity. Therefore, we were unable to account for the difference between light and moderate or vigorous activity. Further examination is needed to better understand the role of travel time, modes of transportation, diversity among the rural population, and physical activity levels. Future studies should also consider using survey instruments to assess health literacy levels.

Our findings underscore the need to develop strategies to improve attendance in CDSMP and DSMP among adults aged 50 or older. Ideally, workshop sites should be located near to participants’ homes to promote completion and increase attendance among urban dwellers. Classes with a larger number of participants should be a goal, keeping in mind program fidelity. Recruitment and program materials should be developed to appeal to people who may not have a high school diploma.
